# Psoriasis Regression Analysis of MHC Loci Identifies Shared Genetic Variants with Vitiligo

**DOI:** 10.1371/journal.pone.0023089

**Published:** 2011-11-18

**Authors:** Kun-Ju Zhu, Yong-Mei Lv, Xian-Yong Yin, Zai-Xing Wang, Liang-Dan Sun, Su-Min He, Hui Cheng, Da-Yan Hu, Zheng Zhang, Yang Li, Xian-Bo Zuo, You-Wen Zhou, Sen Yang, Xing Fan, Xue-Jun Zhang, Feng-Yu Zhang

**Affiliations:** 1 Institute of Dermatology and Department of Dermatology at No.1 Hospital, Anhui Medical University, Hefei, Anhui, China; 2 The State Key Laboratory Incubation Base of Dermatology, Hefei, Anhui, China; 3 The State Department of Dermatology and Skin Science, University of British Columbia, Vancouver, British Columbia, Canada; Oklahoma Medical Research Foundation, United States of America

## Abstract

Psoriasis is a common inflammatory skin disease with genetic components of both immune system and the epidermis. PSOR1 locus (6q21) has been strongly associated with psoriasis; however, it is difficult to identify additional independent association due to strong linkage disequilibrium in the MHC region. We performed stepwise regression analyses of more than 3,000 SNPs in the MHC region genotyped using Human 610-Quad (Illumina) in 1,139 cases with psoriasis and 1,132 controls of Han Chinese population to search for additional independent association. With four regression models obtained, two SNPs rs9468925 in *HLA-C/HLA-B* and rs2858881 in *HLA-DQA2* were repeatedly selected in all models, suggesting that multiple loci outside PSOR1 locus were associated with psoriasis. More importantly we find that rs9468925 in *HLA-C/HLA-B* is associated with both psoriasis and vitiligo, providing first important evidence that two major skin diseases share a common genetic locus in the MHC, and a basis for elucidating the molecular mechanism of skin disorders.

## Introduction

Psoriasis [MIM#177900] is a T cell-mediated inflammatory skin disease, characterized by epidermal hyperproliferation and dermal inflammation [1], [2]. It affects 2–3% of people in the European ancestry population [1], while 0.123% of individuals in the Asian population [3]. Although some advances have recently been made in elucidating the molecular mechanism of psoriasis, its pathogenic mechanism is not completely understood. It is believed that psoriasis has a strong genetic basis, and environmental factor may trigger the initiation of the disease [4].

Over past years, certain efforts have been made to study the genetic basis of psoriasis. Genome-wide linkage analyses have identified nine susceptibility loci (PSORS1–9), only one locus has been consistently replicated. A meta-analysis of multiple genome-wide scans reveals genetic linkage to the major histocompatibility complex (MHC) on chromosome 6p21 that includes the PSORS1 locus, which spans an approximate 220-kb segment on 6p21.3 [5]. The PSORS1 locus is likely to account for about 30% to 50% of the heritability of the disease [6], [7], [8], and has been believed to be the major genetic determinant of psoriasis [9].

The MHC locus is one of the most extensively studied regions in the human genome. Large-scale genetic association studies have identified multiple genetic variants at this locus contributed to risk of a cluster of genetically complex diseases including multiple sclerosis (MS), Type 1 diabetes (T1D), systemic lupus erythematosus (SLE), ulcerative colitis (UC), Crohn's disease (CD), and rheumatoid arthritis (RA) [10]. Recent studies show that MHC loci are likely to play some important roles in psoriasis and vitiligo [11], [12], [13], [14]. The classical MHC locus encompasses approximately 3.6 megabase pairs (Mb) on 6p21.3 and is divided into three subregions: the telomeric class I, class III, and the centromeric class II regions. It has been recently established by the evidence that both linkage disequilibrium (LD) and MHC-related genes exist outside the classically defined locus [15].

Genome-wide association study (GWAS) demonstrates that SNPs in the MHC region are strongly associated with psoriasis in different populations [11], [14]. Several SNPs are associated with psoriasis, but it is still unknown how many independent SNPs located within the MHC region contribute to the risk of psoriasis. The development of psoriasis is believed to involve a major locus PSOR1 in the MHC region and likely be in conjunction with multiple non-MHC loci with common alleles [16]. Since MHC loci have been strongly associated with the development of psoriasis, identification of non-MHC loci associated with psoriasis may have been hindered by likely occurrence of genetic heterogeneity [9]. In addition, a possible reason for the erratic replications of genetic association findings could be that the large effect of the PSORS1 locus (6p21) may affect the effect of other loci involved in psoriasis [5]. Therefore it is necessary to examine the genetic loci associated with psoriasis conditioning on the effect of the PSOR1 locus.

Because of the extensive LD existing between the SNPs within MHC, identification of genetic variants to be associated with human disease is a challenging task. Routine haplotype analysis has a limited role in identifying independent SNPs in such a large linkage disequilibrium block within MHC. Conditional analysis approach adjusting for one top association signals from MHC have been used to search for other independent associations under an additive model [17], [18], [19]. Since a number of association signals are often seen in the MHC region, selections of the top associated SNP for a conditional analysis can vary, consequently may lead to different results.

In this study, we have employed a sophisticated approach to search for independent association signals within the MHC region. We first determined the variable importance of each SNP in the MHC region using both RandomForest algorithm and single SNP association, and then used each of the most important SNPs as a starting SNP to build a multiple regression model from more than 3,000 SNPs within the MHC. In the four regression models we built, two loci in *HLA-C/HLA-B* and *HLA-DQA2* consistently appear in all models, and more importantly, rs9468925 in *HLA-C/HLA-B* is reported as the top association signal in a GWAS of vitiligo in the same population of study. Association analysis of non-MHC loci while controlling for all SNPs in regression models confirms that the association of psoriasis with the *LCE* gene cluster previously identified through a GWAS, and indicates a number of novel non-MHC loci to follow up for further study.

## Materials and Methods

### Study subjects

Study subjects comprised of psoriasis cases and healthy controls recruited across China. Diagnosis, clinical assessment and recruitment of participants have previously been described elsewhere [11]. Briefly, the study subjects were recruited from Chinese Han population through the collaboration by multiple hospitals in China. The clinical diagnosis of all patients was confirmed by at least two dermatologists. Clinical information on patients was collected through a full clinical checkup, and additional demographic information was also collected from both patients and controls through face to face interview with a structured questionnaire. All controls used in this study were individuals who were confirmed without psoriasis, any autoimmune disorders, systemic disorders, and any family history (including first-, second- and third-degree relatives) of psoriasis. Cases and controls were matched by gender and age (within five years). Cases were predominantly early onset patients. The total number of subjects for the analysis includes 1,139 cases with psoriasis and 1,132 controls.

The study was approved by the Ethical Review Board of Anhui Medical University in accordance with the provisions of the Declaration of Helsinki, and by the Institutional Review Board of Anhui Medical University. Collection of blood samples and clinical information from cases and controls was undertaken after a written informed consent was obtained from all participants.

### SNP genotyping

Genotyping has also been described in our previous study [11]. In brief, the genomic DNA was extracted using established method. EDTA anticoagulated venous blood samples were collected from all participants. The genomic DNA was extracted from peripheral blood lymphocytes by standard procedures using Flexi Gene DNA kits (Qiagen) and was diluted to the concentration of 50 ng/µl for the genome-wide genotyping. Samples were genotyped using Illumina Human 610-Quad BeadChips (San Diego, USA). Genome-wide genotype data were filtered sequentially using minor allele frequency>1%, genotyping rate >95%, sample genotyping rate >98% and HWE test p value <0.00001 in the control cohort. After genome-wide quality control of genotype, 3,033 SNPs within a 3.6 Mb of MHC region were included for analysis.

### Statistical analysis

The variable importance was first assessed for all SNPs in the MHC. Traditionally, variable importance of a SNP is determined by p value from single SNP association test, which is based on a statistics of relative measure of estimated coefficient and its standard error. In genetic association study, p value usually measures the linear (or log-linear) relationship between SNP and an outcome of disease. However, the association p value may not measure the importance of a SNP that has both association and possible interactions with other SNPs. Therefore we also applied RandomForest algorithm to determine the variable importance of each SNP in the MHC region [20], [21].

The most important SNPs were selected as starting SNPs to build multiple regression models using stepwise logistic regression. Multiple regression analysis is more powerful in establishing more robust association than single SNP association analysis, because it controls for potential genetic heterogeneity, confounding factors and population stratification [22], [23]. Stepwise regression is an automated procedure that is normally used to select independent variables to build a multiple regression model from a large number of variables. We coded each SNP as a categorical variable (0, 1 and 2). Stepwise logistic regression analysis was performed while controlling for sex, sample geographic location and one of most important SNPs according to both RandomForest analysis and single SNP association. We used the level of 0.05 for entering a SNP, and 0.1 for a SNP being removed from a model. Predicted probability of being in disease group was estimated for each of final logistic regression models, and was used as a covariate for genome-wide association analysis of non-MHC SNPs. The analysis was performed using SAS and Random Forest in R package (http://www.r-project.org/).

## Results

Total 1,139 cases with psoriasis and 1,132 normal controls were included in the analysis (**Table 1**). The ratio of male to female in the study subjects was matched well between cases (669/470) and controls (670/462). The mean age in cases was 30.98 years roughly matched 35.09 years in controls. In the previous GWAS, we have confirmed strong association signals at rs1265181 in the MHC region and, rs3213094 in *IL12B*, and identified several novel susceptible loci in the *LCE* gene cluster on 1q21.

**Table 1:** Summary demographics of study sujects.

	CASES	CONTROLS
sample size	1139	1132
mean age	30.98 years	35.09 years
SD	12.31 years	13.2 years
Male	669	670
Female	470	462

**Figure 1:**
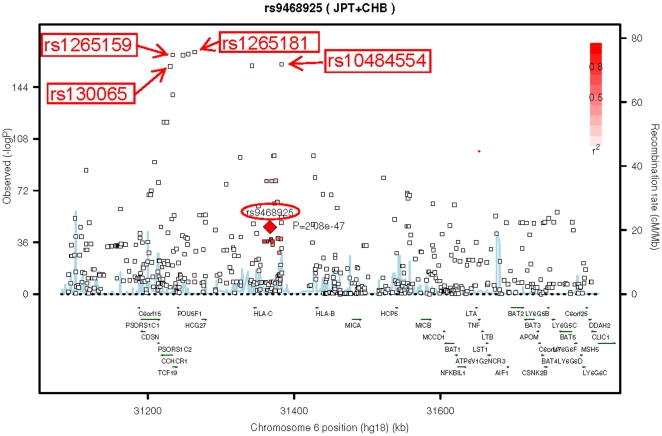
Recombination rates and p values from single SNP associations at four starting SNPs and rs9468925 in *HLA-C/HLA-B* appeared in four models, based on combined Japanese and Han Chinese sample in HapHap data(JPT+CHB) and is not in LD with all of four starting SNPs.

Eight SNPs were found to be the most important in the MHC region using the RandomForest algorithm (**Figure S1**). These included rs130065, rs720465 and rs3130455 in *CCHCR1*, rs1265159 and rs3130457 in *POU5F1*, rs1265181 in *HCG27*, and rs2248902 and rs10484554 in *HLA-C/HLA-B*. Almost all of them were located within the PSOR1 locus and appeared to be in strong LD. The variable importance of SNPs determined through RandomForest algorithm was largely consistent with single SNP association results (**Table S1**). This is expected because the SNPs in the PSOR1 locus tend to have a strong main-effect. Finally SNPs rs130065 (*CCHCR1*), rs3130457 (*POU5F1*), rs1265181 (*HCG27*) and rs10484554 (*HLA-C*) each of them was used as a starting SNP to build a multiple regression model (**Figure 1**). Also these SNPs have previously been associated with psoriasis.

SNPs selected into the multiple regression models were identical between Model 1 and Model 2 except the starting SNPs (**Table 2**). When rs1265181 in *HCG27* was controlled (Model 1), four SNPs including rs9468925 in *HLA-C/HLA-B*, rs2442719 in *HLA-B*, rs4947324 in *NFKBIL1* and rs2858881 in *HLA-DQA2* were selected. While rs130065 in *CCHCR1* was controlled (Model2), the exact same four SNPs were selected, and the effect size for each SNP was very close between two models. As it is expected, both rs1265181 and rs130065 showed much stronger effects than others on the risk of psoriasis in the regression models. We also performed functional prediction of SNPs that were selected into the multiple regression models. Only rs130065 appeared to be non-synonymous, and was found to have regulation potential (prediction score = 0.501) and likely conserved in multiple species (prediction score = 0.348). According to the analysis of genotype data in this study, rs130065 (r^2^ = 0.93, D′ = 0.99) and rs1265181 (r^2^ = 0.99, D′ = 1) are highly correlated with rs3130457, a SNP in *POU5F1* that is completely tagged to the classical HLA-C*0602 allele that has been consistently associated with psoriasis in multiple populations.

**Table 2:** Multiple logistic regression models of MHC Loci.

					Association in GWAS				Multiple logistic regerssion model					
Genename	SNPID	SNP	BP	A1	F_A	F_U	A2	P	OR	95% CI		P_add	P_geno	
**Model 1**														
HCG27*	1344	rs1265181	31263764	G	0.46	0.10	C	5.8E-159	14.04	10.79	18.27	5.65E-86	2.70E-82	
HLA-C	1442	rs9468925	31366816	A	0.21	0.41	G	9.3E-50	0.65	0.54	0.79	1.70E-05	1.14E-05	**
HLA-B	1499	rs2442719	31428517	A	0.64	0.34	G	3.0E-92	1.61	1.30	2.01	1.66E-05	5.58E-06	
NFKBIL1	1752	rs4947324	31636109	T	0.13	0.05	C	1.3E-21	2.20	1.62	2.99	4.83E-07	8.85E-05	
HLA-DQA2	2476	rs2858881	32811823	G	0.04	0.04	A	7.3E-01	1.91	1.27	2.89	2.08E-03	7.42E-03	**
**Model2**														
CCHCR1*	1301	rs130065	31230479	T	0.48	0.11	C	9.6E-170	11.22	8.73	14.43	2.65E-79	4.86E-82	
HLA-C	1442	rs9468925	31366816	A	0.21	0.41	G	9.3E-50	0.62	0.51	0.75	1.13E-06	2.40E-05	**
HLA-B	1499	rs2442719	31428517	A	0.64	0.34	G	3.0E-92	1.65	1.33	2.05	5.28E-06	3.24E-05	
NFKBIL1	1752	rs4947324	31636109	T	0.13	0.05	C	1.3E-21	2.15	1.59	2.91	7.01E-07	9.54E-06	
HLA-DQA2	2476	rs2858881	32811823	G	0.04	0.04	A	7.3E-01	1.72	1.13	2.63	1.18E-02	5.23E-03	**
**Model 3**														
OR12D2	49	rs9257843	29476644	C	0.49	0.38	T	8.7E-13	1.34	1.13	1.58	7.10E-04	5.54E-04	
IER3	912	rs9262176	30839309	T	0.02	0.01	C	8.1E-02	2.76	1.41	5.38	2.99E-03	1.99E-03	
POU5F1*	1328	rs1265159	31248026	T	0.46	0.10	C	1.2E-156	24.31	18.47	32.00	1.26E-114	2.39E-109	
HLA-C	1442	rs9468925	31366816	A	0.21	0.41	G	9.3E-50	0.53	0.43	0.66	1.22E-08	7.75E-08	**
LOC729816	1522	rs7770216	31448590	T	0.36	0.39	G	5.1E-02	1.90	1.53	2.37	9.01E-09	8.82E-08	
MICA	1554	rs2251396	31472686	T	0.15	0.25	C	1.1E-18	0.24	0.16	0.37	7.62E-11	2.53E-10	
MICA	1556	rs2523454	31475844	T	0.20	0.28	C	8.3E-12	3.28	2.04	5.29	1.03E-06	5.86E-07	
MICA	1566	rs12175489	31485566	A	0.19	0.31	G	6.0E-20	0.59	0.43	0.82	1.36E-03	2.87E-04	
NOTCH4	2080	rs511027	32314665	A	0.06	0.08	T	6.9E-03	1.52	1.09	2.14	1.49E-02	1.82E-04	
HLA-DQA2	2476	rs2858881	32811823	G	0.04	0.04	A	7.3E-01	1.81	1.21	2.71	3.66E-03	1.53E-03	**
COL11A2	2922	rs9368758	33245999	A	0.47	0.51	G	5.9E-03	1.33	1.11	1.59	1.71E-03	1.25E-03	
**Model 4**														
OR12D2	49	rs9257843	29476644	C	0.49	0.38	T	8.7E-13	1.29	1.09	1.53	3.26E-03	2.10E-03	
IER3	912	rs9262176	30839309	T	0.02	0.01	C	8.1E-02	3.11	1.57	6.16	1.12E-03	9.98E-04	
POU5F1	1340	rs7760698	31258204	A	0.01	0.02	T	1.2E-01	0.23	0.09	0.59	1.93E-03	2.76E-03	
HCG27	1344	rs1265181	31263764	G	0.46	0.10	C	5.8E-159	4.68	2.13	10.31	1.28E-04	6.19E-07	
HLA-C	1442	rs9468925	31366816	A	0.21	0.41	G	9.3E-50	0.58	0.47	0.73	2.03E-06	9.03E-06	**
HLA-C*	1483	rs10484554	31382534	T	0.48	0.11	C	5.5E-158	6.35	2.87	14.06	5.12E-06	3.20E-05	
LOC729816	1522	rs7770216	31448590	T	0.36	0.39	G	5.1E-02	1.74	1.40	2.17	8.61E-07	6.79E-06	
MICA	1554	rs2251396	31472686	T	0.15	0.25	C	1.1E-18	0.24	0.16	0.37	1.18E-10	2.61E-10	
MICA	1556	rs2523454	31475844	T	0.20	0.28	C	8.3E-12	3.84	2.36	6.23	5.49E-08	7.01E-08	
MICA	1566	rs12175489	31485566	A	0.19	0.31	G	6.0E-20	0.62	0.45	0.86	4.20E-03	2.35E-03	
NOTCH4	2080	rs511027	32314665	A	0.06	0.08	T	6.9E-03	1.47	1.05	2.08	2.66E-02	2.27E-04	
HLA-DQA2	2476	rs2858881	32811823	G	0.04	0.04	A	7.3E-01	1.98	1.31	2.98	1.08E-03	3.45E-04	**
COL11A2	2922	rs9368758	33245999	A	0.47	0.51	G	5.9E-03	1.36	1.13	1.62	9.52E-04	1.67E-03	

Note:

**are SNPs consistently appeared in 4 models; highlighted are starting SNPs in each model; sex and sample region were controlled in each model; the first SNPs forced to be in the model.

Majority of SNPs selected in Model 3 (*POU5F1*) and Model 4 (*HLA-C*) was similar (**Table 2**). SNPs rs9257843 in *OR12D2*, rs9257843 in *IER3*, rs9468925 in *HLA-C/HLA-B*, rs7770216 in *LOC729816*, rs2251396, rs2523454 and rs12175498 in *MICA*, rs511027 in *NOTCH4*, rs2858881 in *HLA-DQA2* and rs9368758 in *COL11A2* appear to be significant in Model 3. In addition to the same set of SNPs, two additional SNPs–rs7760698 in *POU5F1* and rs1265181 in *HCG27* were selected into Model 4. The starting SNP rs3130457 at *POU5F1* completely tags to the classical HLA-C*0602 in Han Chinese, and it is in strong LD with another starting SNP rs10484554 (r^2^ = 0.9, D′ = 0.982) in *HLA-C*. It is worth noting that rs1265181 in *HCG27* selected into Model 4 with *HLA-C*, also showed the strongest association in Model 1.

**Figure 2:**
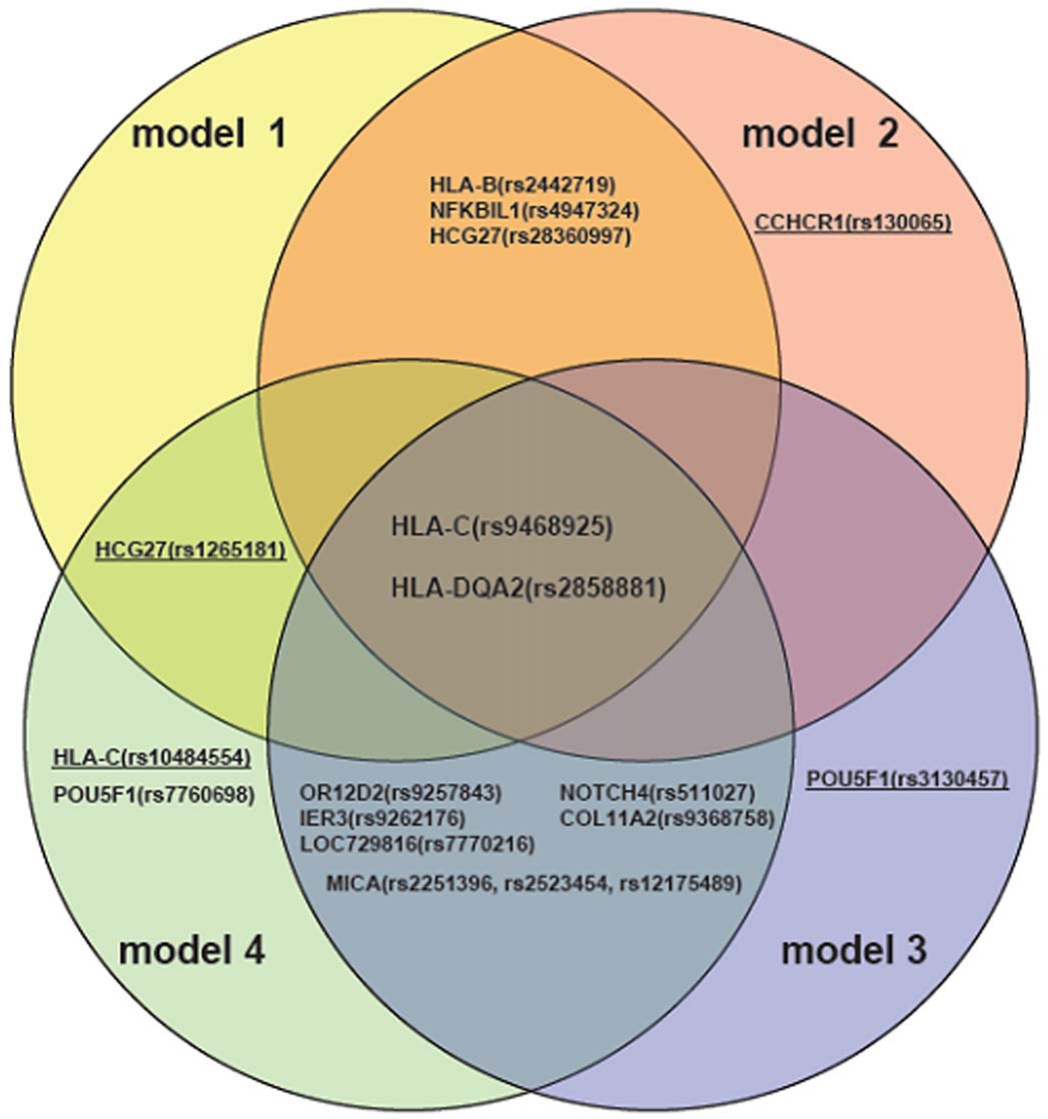
Venn diagram of SNPs selected in four models. Intersection indicates shared SNPs selected from different models.

Very interestingly, two common SNPs in *HLA-C/HLA-B* and *HLA-DQA2* were selected by the entire four regression models (**Table 1** **,** **Figure 2**). Minor allele at rs9468925 in *HLA-C/HLA-B* appear to be negatively associated with psoriasis in all four models (OR = 0.53–0.65, P = 1.22e-08 to 1.70e-05). SNP rs9468925 was clearly independent of the top four SNPs that were used as starting SNPs for building multiple regression models, and neither in LD with the HLA-C*0602 allele (r^2^ = 0.177) in Han Chinese (**Figure 1**). SNP rs2858881 in *HLA-DQA2* (OR = 1.72 to1.98, P = 1.08e-03 to 1.18e-2) were also consistently included in all regression models (**Table 1**), while it is not significant in single SNP association in previous GWAS of psoriasis.

A number of non-MHC loci appeared to be significantly associated with psoriasis while the MHC locus was controlled (**Table S2**). The most significant association of non-MHC SNPs with psoriasis were observed at four SNPs in *LCE3A* and *LCE3D* in the *LCE* gene cluster (p<5e-06), which is consistent with previous findings from GWAS [11]. Fifty-six SNPs in 39 genes were associated with psoriasis. Fifteen out of thirty-nine genes were involved with functions of cell death, cell to cell signaling and interaction, and other fifteen genes were unknown for their functions.

## Discussion

We have examined the association of SNPs in the MHC region with psoriasis using multiple regression models. When the top associated SNPs in PSOR1 locus were controlled, stepwise logistic regressions of 3,033 SNPs from the MHC region largely showed two sets of markers (Model 1, 2 vs Model 3, 4) associated with psoriasis, although starting four SNPs (rs130065, rs3130457, rs1265181 and rs10484554) are in strong LD. SNP rs9468925 in *HLA-C/HLA-B* consistently appears in all four regression models, indicating that an independent *HLA* locus is associated with psoriasis. It is interesting to see that rs9468925 is also the top associated SNP in a recent GWAS in Han Chinese population with vitiligo, another major skin disorder with autoimmune components [24]. We have provided one important evidence for that psoriasis shares the common genetic variants with vitiligo, and the effect size is similar (OR = 0.65 in psoriasis vs. OR = 0.75 in vitiligo).

When top SNPs rs1265181 in *HCG27* or rs130065 in *CCHCR1* is controlled, the same set of SNPs from *HLA-B*, *HLA-C/HLA-B*, *HLA-DQA2* and *NFKBIL1* are selected. This is expected because two SNPs are in strong LD (r2 = 0.935, D′ = 0.998) and both are good approximate to HLA-C*0602, known to be associated with psoriasis. SNP rs1265181 in *HCG27* is the top association signals in a GWAS of psoriasis in Han Chinese population [11], while rs130065 in *CCHCR1* has been consistently associated with psoriasis in multiple populations [25], [26], [27], and *HCR1* protein expressed differently between lesional skin and normal skin [26]. *CCHCR1* may function as a negative regulator of keratinocyte proliferation; aberrant function of *CCHCR1* may lead to abnormal keratinocyte proliferation which is a key feature of psoriatic epidermis [28]. However, other studies show that the association may be due to the strong linkage disequilibrium with HLA-C*0602, and it is difficult to distinguish the genetic association of *CCHCR1* from HLA-C*0602, which is completely tagged by a single SNP (rs3130457, Allele A) in Han Chinese population [29].

While HLA-C*0602 (approximate to rs130065 and tagged by rs3130457) is controlled, multiple regression analysis of SNPs in the MHC loci reveal independent associations of additional both classical *HLA* and non-*HLA* loci in the MHC region with psoriasis. *HLA-DQA2*, functioned as antigen presentation and likely expressed in human B lymphoblastoid cell lines [30], [31], has been associated with autoimmune disorders such as type 1 diabetes, rheumatoid arthritis and alopecia areata [32], [33]. *NFKBIL1*, a non-*HLA* gene, encodes a divergent member of the I-kappa-B family of proteins, critical components of the NF-κB signaling pathway. It regulates the transcription of many important mediators of inflammation and tissue destruction in the psoriasis including *TNF*, *IL-1* and *IL-6*. *NFKBIL1* has also been associated with autoimmune disorders such as rheumatoid arthritis, SLE and Sjogren's syndrome [34], [35]. This suggests that in addition to genetic variants that regulate keratinocyte proliferation, genes involved with autoimmune diseases likely contribute to the risk of psoriasis, supporting that psoriasis has autoimmune components. We have also found that rs2442719 in *HLA-B* is associated psoriasis. This is consistently with the previous analysis, conditioning PSOR1 locus (HLA-C*0602) in both Chinese and European ancestry population [18].

Two similar regression models were built when SNPs rs3130457 in *POU5F1* and rs10484554 in *HLA-C* were controlled. Although two models selected different sets of SNPs from previous ones when SNPs in *HCG27* and *CCHCR1* were controlled, SNPs rs9468925 in *HLA-C/HLA-B* and rs2858881 in *HLA-DQA2* were repeatedly selected, providing robust evidence for that association of these two classical *HLA* loci with the development of psoriasis. SNP rs3130457 in *POU5F1* tags the classical HLA-C*0602 allele, while rs10484554 in *HLA-C* is the most significant SNP associated with psoriasis and psoriatic arthritis in European ancestry population [36]. It is interesting to note that rs3130457 in *POU5F1* is in strong LD with rs1265181 in *HCG27* and rs130065 in *CCHCR1*, but two different models were built. In addition, we found that SNPs from several non-*HLA* genes including rs2251396, rs2523454 and rs12175589 in *MICA*, rs7770216 in *LOC729816*, rs511027 in *NOTCH4*, rs9257483 in *OR12D2* and 9262167 in *IER3* were associated with psoriasis in multiple regression analyses. Except that rs7770216 in *LOC729816* has been reported to significantly interact with rs563495 on rheumatoid arthritis [37], and rs2251396 has been tagged to classical HLA-B*04601 allele in Japanese population, all others non-HLA loci appear to be novel loci for psoriasis.

Although these non-*HLA* loci in the MHC region are statistically less significant than compared to the *HLA* loci, they may shed a light on additional components of pathogenesis in psoriasis. All of these non-*HLA* genes such as *POU5F1*, *MICA*, *NOTCH4*, *IER3* and *OR12D2* are coding for proteins. *POU5F1* is a transcription factor and encodes a key regulator of stem cell pluripotency, and has been noted as one of four genes having stem cell potential and being expressed in embryonic stem cells and tumor cells [38]. *POU5F1* is likely to play a role in epithelial tumors such as skin and salivary glands [39], [40]. Previous studies have shown that a genetic polymorphism of *POU5F1* is associated with psoriasis in Chinese population [41]. A stronger association than HLA-C*0602, in particular in HLA-C*0602 negative individuals, is observed with psoriasis in Spanish population [42]. *MICA* is expressed on the cell surface, and functions as a stress-induced antigen that is broadly recognized by intestinal epithelial gamma delta T cells. Although *MICA* is strongly in LD with *HLA-B*, it has been associated with psoriasis and evidenced for an association independently of *HLA-B/C* with psoriatic arthritis in Jewish population [43], [44]. *NOTCH4* encodes a member of the Notch family that play a role in a variety of developmental processes by controlling cell fate decisions. *NOTCH4* has been predominantly associated with neuropsychiatric disorders [45], [46]; little information is available on the association with psoriasis. *IER3*, an early response gene that is induced by ionizing and ultraviolet radiation, is widely expressed in epithelial and endocrine tissues and the expression is regulated by multiple transcriptional factors such as *NF-kappaB/rel*, p53 and c-Myc [47], [48], [49]. It accelerates cell cycle progression and supports the survival of T cells, causing autoimmune disease and the development of T cell lymphoma. *OR12D2*, olfactory receptors interact with odorant molecules in the nose to initiate a neuronal response that triggers the perception of a smell. Defects in *COL11A2* are the cause of deafness autosomal dominant type 13 (*DFNA13*), form of sensorneural hearing loss, which results from damage to the neural receptors of the inner ear, the nerve pathways to the brain.

Our study may have some important implications for searching for genetic variants associated with complex human disorders. Primary nature of psoriasis is as an epithelial and immunological disorder with autoimmune cause of inflammatory process. Genetic components of both immune system and the epidermis contribute to the disease [50]. Previous single SNP-phenotype analysis may not reflect the nature of parthenogenesis of the disease. Multiple regression analysis allows us to examine how multiple relatively independent genes together contribute to the risk of disease. It is believed that environmental factors play a certain role in the development of psoriasis. Identification of genetic association in those environmental response genes may help to elucidate the molecular mechanism of the disease.

In summary, through multiple regression analysis of SNPs in MHC loci, we found SNPs in classical *HLA* gene shared between two major skin disorders–psoriasis and vitiligo. In addition to classical *HLA* genes such as *HLA-C*, *HLA-B* and *HLA-DQA2*, we also find association of non-*HLA* genes in the MHC region such as *POU5F1*, *NFKBIL1*, *NOTCH4*, *MICA*, *IER3* and *OR12D2* with psoriasis. Our analysis may provide the first genetic evidence that psoriasis is involved with multiple independent components of immune response, inflammation, skin keratinization and proliferation, autoimmune and stress-related pathways. This multimarker analysis may provide a basis for the disease-prediction based on genetic variants associated with the disease.

## Supporting Information

Figure S1 **Variable importance determined by RandomForest alrgoritm.**
(DOC)

Table S1 **Top 8 associated SNPs determined by RandomForest algorithm.**
(XLSX)

Table S2 **Association of Non-MHC SNPs with psoriasis while controlling for multiple regression models of multiple MHC markers.**
(XLSX)
