# Supplementary material for: Psoriasis Regression Analysis of MHC Loci Identifies Shared Genetic Variants with Vitiligo
Source: PLoS One. 2011 Nov 18;6(11):e23089. doi: 10.1371/journal.pone.0023089 (PMC3220662; doi:10.1371/journal.pone.0023089)

**Figure S1. Variable importance determined by RandomForest alrgoritm.**

| ID | Gene | SNP |
| --- | --- | --- |
| s1301 | CCHCR1 | rs130065 |
| s1306 | CCHCR1 | rs720465 |
| s1307 | CCHCR1 | rs3130455 |
| s1328 | POU5F1 | rs1265159 |
| s1337 | POU5F1 | rs3130457 |
| s1344 | HCG27 | rs1265181 |
| s1412 | HLA-C | rs2248902 |
| s1483 | HLA-C | rs10484554 |

**Figure S2. The recombination plot of HLA-DQA2**


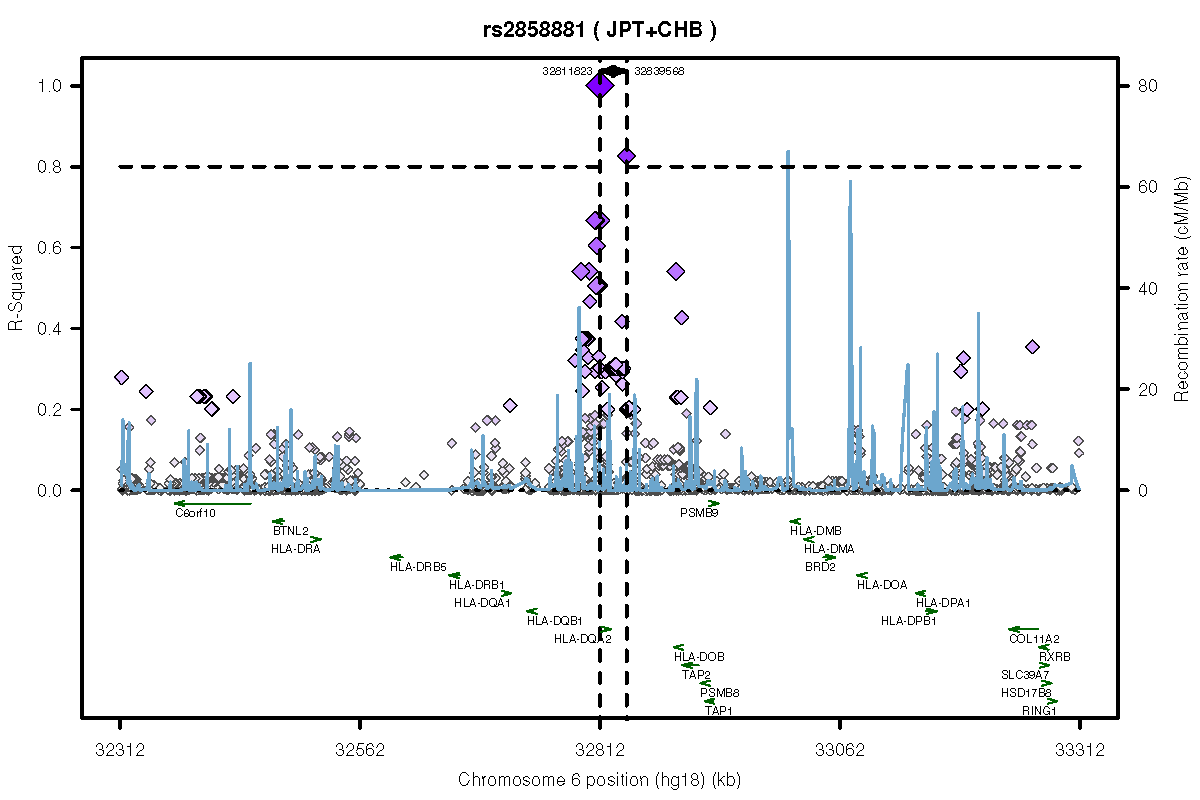

Supplement: Figure S1 — Variable importance determined by RandomForest alrgoritm. (DOC) [file pone.0023089.s001.doc]
